# A Study on the Path of Narrative Renewal of Traditional Villages: A Case of Shawan Ancient Town, Guangdong, China

**DOI:** 10.3390/ijerph20010372

**Published:** 2022-12-26

**Authors:** Zhongwei Wang, Qianda Zhuang, Yue Ma, Pengnan Li

**Affiliations:** 1 School of Art and design, Guangdong University of Finance and Economics, Guangzhou 510320, China; 2 College of Agriculture and Forestry, Linyi University, Linyi 276000, China

**Keywords:** narrative renewal strategy, text construction, narrative clues, Shawan Ancient Town

## Abstract

Traditional villages are a valuable for their historical and cultural heritage, and have long been the focus of academic research regarding their protection and renewal methods in the face of increasing urbanization. In the village renewal model, the continuation and regeneration of cultural context is the core issue. This paper aims to construct the updated narrative text of Shawan Ancient Town on the basis of narrative rules and methods, while also exploring how to generate the conservation and renewal strategies based on the narrative text. Drawing on the concepts and methods of narratology and based on the cultural background and current conditions of Shawan Ancient Town’s architectural heritage, this paper takes “narrative in terms of theme, clue, path, and material” as the basic framework to construct the narrative text in the context of traditional village renewal. Then four aspects, such as “base combing, interface integration, patch symbiosis, and debris collection”, are used to update the methods of the spatial situation of individual places. Additionally, multiple construction forms are explained, generating a historic, interesting, and experiential strategy for the protection and renewal of ancient towns. This study demonstrates logical thinking from the construction of narrative text to the generation of renewal strategies from a narrative perspective while deepening the typical research on the traditional village narrative renewal mode. It is advantageous to build a protection mechanism for historical and cultural villages, and explore a path to protect, inherit, and promote the cultural heritage of villages based on China’s current situation.

## 1. Introduction

Rural areas presently remain the focus of the Chinese government’s attention and policy-led intervention. The No. 1 Central Document in 2013 emphasized increasing efforts to protect traditional villages and dwellings with historical and cultural value and ethnic and regional elements [1]. The No. 1 Central Document for 2017–2018 successively proposed strategies for developing pastoral complexes and rural revitalization [2]. With the development of rural construction activities, the direction of government-led rural construction has begun to change from a single mode of urban–rural integration to a composite mode of protecting and revitalizing of traditional villages. Meanwhile, the appearance of these villages has been significantly improved. However, a considerable number of villages still exhibit the phenomenon of urbanization and convergence due to rapid urban expansion [3]. Moreover, constructive damage is caused due to the misreading of rural landscapes.

The preservation and revitalization of traditional villages have long been a hot topic in rural research in China. The “circle layer” early protection planning model [4] and the holistic protection model rooted in the local area have been proposed [5], as well as the authenticity renewal model based on “the inheritance and survival of rural historical authenticity pattern” [6], and the organic renewal model based on “the scattered integration of historical elements” [7,8]. However, the aforementioned research focuses on the principles of texture repair and overall style shaping for historical villages, and there is still a lack of new perspectives and methods on the inheritance of village context.

Except for China, more research in this field have been carried out worldwide. Cullen (1964) discussed a series of experiences in exploring urban design based on people’s perception of urban space [9]. He revealed the idea of building urban environment based on the behavior of rural subjects, which provided a reference for rural renewal. Potteiger and Purinton (1988) brought the concept of narrative to the field of landscape design in Landscape Narratives: Design Practices for Telling Stories. They used landscapes to tell historical stories and awaken collective memory through various narrative strategies such as naming, sequence, revealing, hiding, agglomeration, and opening, and established a theoretical framework based on the elements, processes, and forms of landscape narratives [10]. The aforementioned study on the concept of time–space analysis and landscape narrative logic has established an important foundation for the rural narrative framework.

After summarizing the experience, methods, and regulations of small town planning and design in the suburbs of metropolitan cities in the United States, Randall Arendt proposed the protection planning path of towns and villages which combined nature and culture [11], further establishing a methodological foundation for the continuation of the rural context. Interactive studies on narratology and spatial design began in the 1980s, providing new insight for this study. In 1985, Hejduk began advocating the intersection of narrative and art [12], whereas in 1986, Foucault extended the conceptual approach of narrative to other disciplines through the analytical thinking of simultaneous time and space [13], from which the concept of the spatial narrative was derived. 

Bacon and Cullen proposed synchrony correlation strategies for many places, such as sequence view and field connection lines [14,15]. Azaryahu used urban relics in North America, Europe, and Israel as examples to explore the narrative construction path of historical blocks and proposed three spatial narrative strategies, namely, declarative, continuous or discontinuous, and subject narrative [16]. These studies focus on synchronic strategy research, but the discussion on the diachronic strategy of place is limited.

In conclusion, narratology is introduced into place theory, coupling the material space structure of places and their cultural significance; effectively organizing the historical characteristics, perceptual experience, and cultural information of the site and its settlers together; and establishing a new perspective for the shaping of current urban cultural characteristics [17]. However, there is a lack of more multidimensional and new interpretations of this topic because of the relatively single perspective of the research. Current research on spatial narrative focuses more on the “construction of narrative space” or “the setting of plot nodes” in the field of urban renewal and architectural design [18], with few narrative studies in the fields of rural renewal. Furthermore, more theoretical research on spatial narrative is performed than practical application, and correlation integration research is explored on the basis of the case of rural clues. Therefore, this paper draws on the rules and methods of narratology to construct narrative texts, determine the clues of place design, arrange narrative plots, realize the construction of space elements and context reconstruction, and finally, achieve the research objective of making rural renewal strategies more historical, interesting, and empirical. Specifically, our research questions are as follows: 

RQ1: How is the narrative text constructed on the basis of narrative theory and the current conditions of Shawan Ancient Town?

RQ2: How are the conservation and renewal strategies of Shawan Ancient Town constructed on the basis of narrative theories and texts?

## 2. Research Methodology

### 2.1. Case Study 

**Figure 1:**
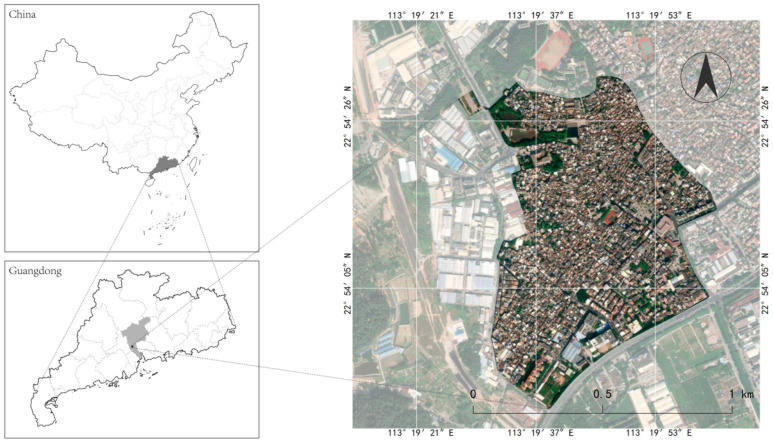
Location of Shawan Ancient Town in Guangzhou, Guangdong. (Source: the authors).

**Figure 2:**
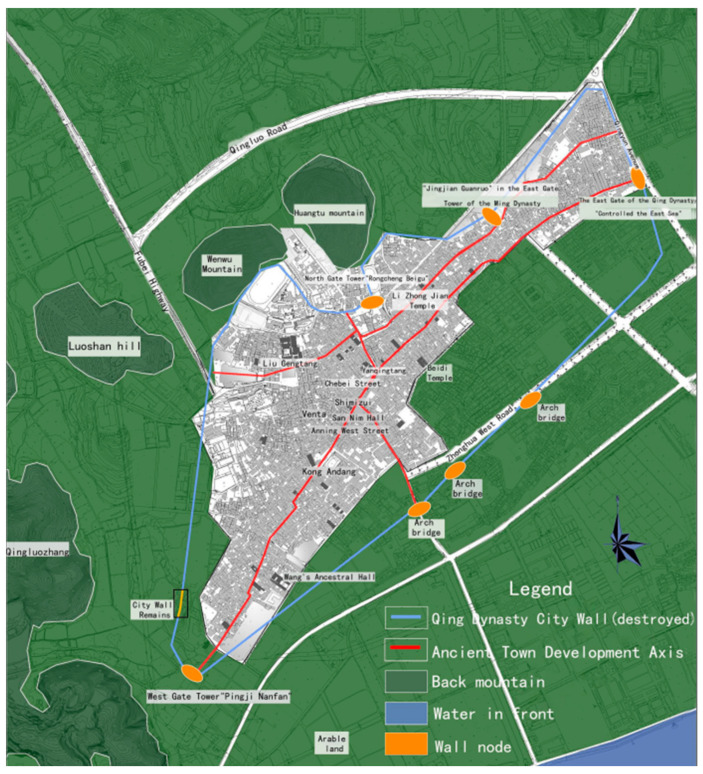
Analysis map of the external environment of Shawan Ancient Town. (Source: [20]).

**Figure 3:**
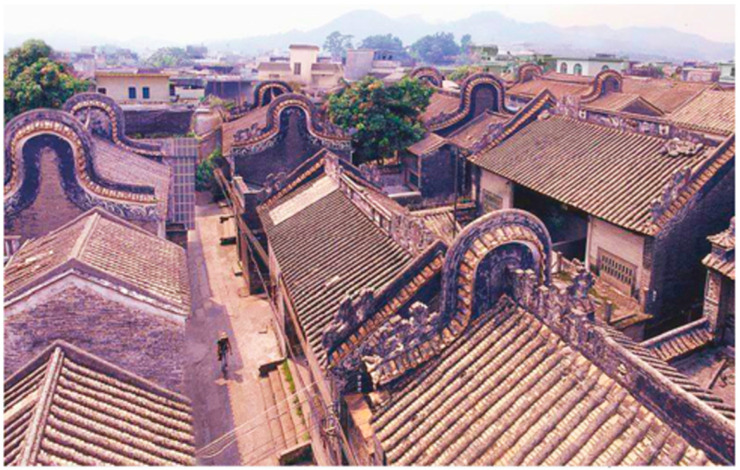
Shawan buildings (Source: the authors).

The Shawan Ancient Town is located in Panyu District, Guangzhou, near the Pearl River Estuary (Figure 1). The ancient town covers an area of 53.72km ² and includes 16,710 households with 56,077 people [19]. It consists of Shawan East Village, Shawan West Village, Shawan North Village, and Shakeng Village. The terrain is wide from east to west, narrow from north to south, and slopes from north to south. It is an 800-year-old traditional town in the Pearl River Delta, founded during the Southern Song Dynasty. The sandy bay area was a small sand island in ancient times, which gradually silted up and expanded after the long-term alluvial of the seawater until a half-moon-shaped platform surrounded by Qingluozhang and surrounding hills were formed during the Republic of China period [6]. Therefore, it is surrounded by water, with the Shiqiao Waterway and Shawan Waterway to the north and the Zini River Waterway to the west. Additionally, it is close to Wenwu Mountain and Huangtugang to the north and Qingluozhang Mountain to the west, forming a subtropical Lingnan water town pattern with mountains behind and surrounded by rivers and crisscrossing river networks (Figure 2). Shawan Ancient Town has about 7 hm^2^ of ancient buildings, with a total of 49 cultural relics, including 1 provincial cultural relic protection unit, 2 municipal cultural relic protection units, 7 municipal registered cultural relics, and 39 district registered cultural relics [19]. There are a significant number of Cantonese traditional houses in the Shawan Ancient Town, such as bamboo tube houses, three-room and two-corridor houses, Ming Dynasty-style houses, and Western-style houses. There are more than 50 ancient ancestral halls. Meanwhile, there is a huge amount of intangible cultural heritage, such as He’s elegant music, Shawan lion-awakening dance, brick carving, gray carving, stone carving, and the gray sculpture genre. Therefore, Shawan is known as the “Hometown of Guangdong Music” and “Hometown of Folk Sculpture” (Figure 3).

**Figure 4:**
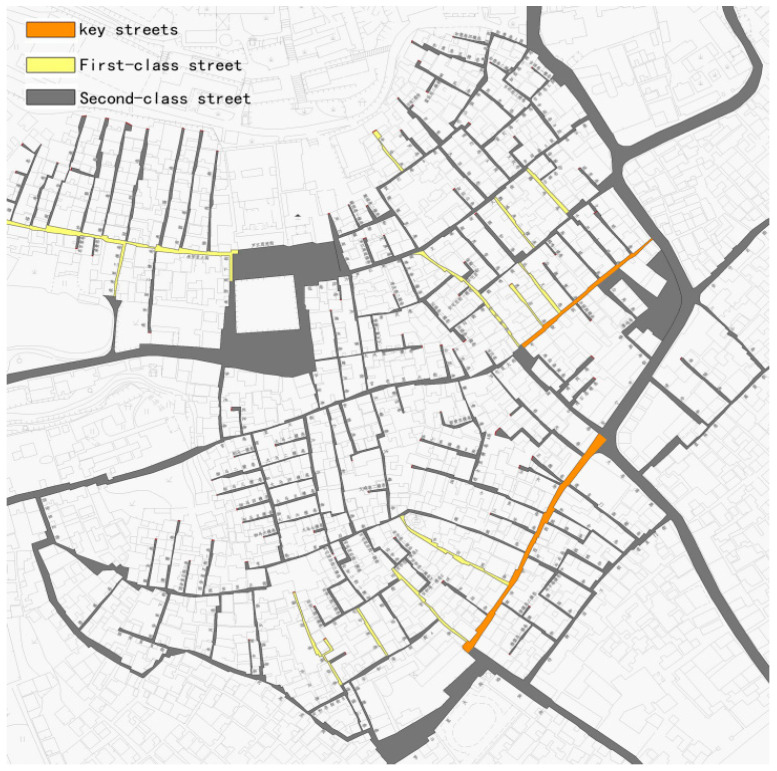
Pattern and texture of Shawan Ancient Town. (Source: [20]).

The Shawan Ancient town is composed of the He Clan, the Wang Clan, and other clans, and its layout is centered on Liugengtang. The rest of the buildings expand outward in a fan shape. The streets run vertically from north to south, forming cold alleys. It builds a crisscross pattern with three main horizontal streets, namely, Wenlinfang, Guanxiangli–Guanzhaili, and Anning West Street. Anning West Street in the south and the nearby Daxiangyong Road constitute a group of residential buildings with commercial characteristics, contributing to a typical commercial town pattern of “three streets and six cities” in central Guangdong (Figure 4).

With the intensification of urbanization, all types of self-built tile houses have emerged one after the other and numerous rural landscape relics have become increasingly abandoned. The historical context and texture of this town have been dramatically affected. Therefore, reconsidering the continuity of cultural context and practical functional needs, and balancing the relationship between traditional features and tourism development are the primary issues for the protection and renewal of Shawan Ancient Town.

### 2.2. Research Method

This paper aims to explore a new path for the protection and renewal of traditional villages in the transformation of times from the perspective of narratology. Therefore, it is necessary to introduce research cases, data collection, and analysis, and to reveal results. This study adopts mixed qualitative research method. The basic methodology used is case studies [21,22]. Through the case study, the understanding of rural spatial cognition and rural conservation and renewal path is obtained in the context of narratology.

(1) Data collection and renewal practice

On the basis of theoretical literature research, the author participated in the protection and renewal process of Shawan Ancient Town and recorded the measures and problem-solving methods in the renewal process. After the completion of the project, from January 2020 to September 2022, the author kept track of the use of the project, made several visits to the ancient village, conducted field research such as photographing and mapping, and took more than 100 photos as basic data.

(2) Data Analysis

The empirical part of this study combines qualitative case analysis with narrative method. In order to obtain qualified data for research, this paper adopts the method of purposive sampling to select representative site nodes and images for analysis. The characteristics of rural pattern and spatial interface are analyzed by means of iterative fractal analysis, multivariate comparison, and structural analysis. After the collected photos regarding to the Shawan Ancient Town were prepared, the QSR Nvivo 12 software was used to encode the photos based on their display content [23]. Through the coding process, a catalogue was obtained, thereby revealing the results of the building type modification in Section 4.

## 3. Narrative Text Construction

### 3.1. Narratology Methods and Text Construction Ideas

Narratives are basic ways and channels to deliver information, expression, and communication. In short, it is “telling a story” [24]. Spatial narrative generally includes four aspects: “narrative theme, narrative clue, narrative path, and narrative material”. Narrative logic has four types: sequence, interstitial, skipping, and flashback narrations. The narrative process mainly consists of a prologue, beginning, development, climax, ending, and epilogue [25].

In terms of narrative clues, Lin explained the effect of multiple narrative clues on the renewal of the historical environment [18]. While Dai (2019) proposed a design translation path according to the narrative methods in literature and art, such as “collage”, “reproduction” and “symbol” [26], which enrich the narrative skills of the non-material elements of the place.

On the basis of UNESCO’s recognition of the attributes and protection principles of historical and cultural villages and towns, traditional village narrative texts run through two basic levels. One is the value association of “space and culture” [27], that is, “the morphological relationship and organizational relationship between the historical built-up area of a town and its rooted regional environment, as well as various landscape heritage objects” [28]. The other level is the historical stratification of “time and memory”, namely, the memory accumulation presented by “the continuous superposition and accumulation of historical relics of different periods in a town on a specific spatial range or spatial object” [29].

**Figure 5:**
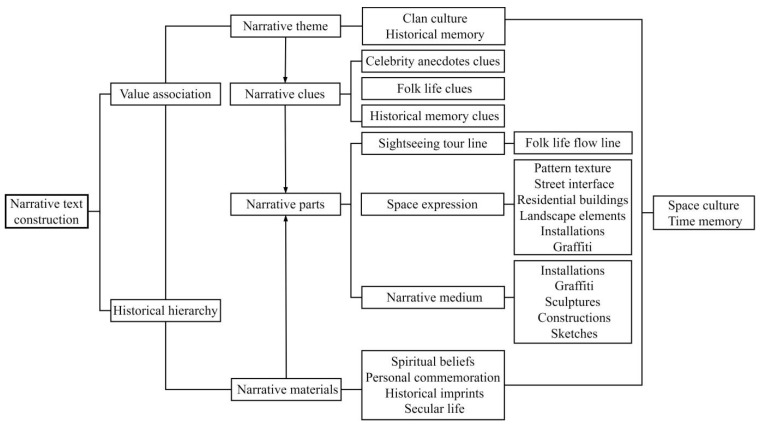
Construction of narrative text. (Source: the authors).

The village renewal narrative emphasizes the new path to preserve local culture and historical memory (Figure 5). Specifically, it starts with value correlation and historical stratification. Then, the contextual connection of rural places and the reproduction of historical memory are completed through the pursuit of a series of hidden clues and the creation of node situations along the multilevel narrative path.

The context of the place includes factors such as folk customs, beliefs, and clans. Historical memory emphasizes hidden stories in historical space or topics that can be reproduced by retaining fragments. Narrative materials are extracted from settlement folk culture, religious beliefs, celebrity anecdotes, and historical memories, and then narrative clues are generated to explain historical stories and awaken the collective memory.

In rural renewal, the narrative path is expressed at three levels: “narrative media, spatial expression, and viewing streamline”. Narrative media include different means of expression, such as installations and graffiti. The viewing streamline involves the design of one or more important flow lines, as well as the staged planning of the beginning, development, climax, and end of the flow line. The final narrative is displayed in the creation of an interface form with the space path as the carrier based on multiple levels of pattern texture, street and alley interface, residential buildings, and landscape elements.

### 3.2. Narrative Theme and Clues

Place context includes folk customs, beliefs, clans, and other factors, whereas historical memory focuses on hidden stories in historical space or topics that can be reproduced because of retained fragments. Therefore, the narrative text takes “the inheritance and continuation of Shawan in the local context” as its theme and extracts local narrative themes and clues from four aspects, such as “spiritual belief, character commemoration, secular life, and historical relics” (Table 1) to elaborate historical stories and arouse collective memory.

On the basis of the aforementioned content, a narrative structure with multiple clues juxtaposed in three-dimensional space is constructed as follows:

- Religious Culture clues: Liugengtang, Yuxu Palace, and Wenfeng Pagoda are taken as the central nodes to interpret the belief culture of Shawan Ancient Town.
- Folk culture clues: on the basis of the themes of Shawan music, floating colors, and folk games, the San Nim Hall Folk Museum and landscape sculptures are built to express the local folk culture. They also include celebrity anecdotes, such as the old residences and houses of He Shaoxia, He Binglin, and other celebrities, combined to build a cultural memorial site in the Shawan Ancient town.
- Historical memory clues: with the historical memory of the village as a narrative blueprint, the core node is to explore memory sites with a sense of historical footage. Simultaneously, corridors and paths are used to connect multiple core groups, and linear landscapes continue the “history-modern” time memory image. The Lujiang Ancient Road Wall and Qingshuijing Square are established to illustrate the story of Shawan’s historical changes, along with historical relics, such as the ancient walls of streets and lanes and Qingshui well (Figure 5).

### 3.3. Narrative Path and Media

The narrative path is completed through “narrative media, spatial expression and viewing streamline”. Narrative media involve devices, graffiti, and other expression means. The viewing streamline involves one or more important streamline and the staged planning of the beginning, development, climax, and end. Finally, narrative takes the space expression as the carrier, and relies on the pattern texture, street and lane interface, residential buildings, and landscape elements to show in the interface construction.

Following narrative clues and material planning and viewing sequences, the narrative path is completed through “level sorting of pattern bases, morphological integration of street and alley interfaces, memory regeneration of space patches, and fragmentation of node elements”. The fragmentation of node elements and the alienation and superposition of fragment forms are used to highlight the richness of spatial experience between multiple attraction points and to reconstruct a recessive expression sequence on the old base site. Meanwhile, multinarrative media of modern art present characteristics of rural situations, such as landscape installations with rural themes, painted graffiti, and other themed items, generating synchronic expressions under different discourse concepts in the objective environment.

## 4. Narrative Updating Strategy for Shawan Ancient Town

The background-style field base is frequently embodied in a certain form of spatial configuration, which is required for the horizontal reading of field narratives [30]. Different coordination methods are formed by differences in the types of centers and edges for the scattered village bases and interfaces.

### 4.1. Defining the Planning Scope and Clarifying the Protection Level

**Figure 6:**
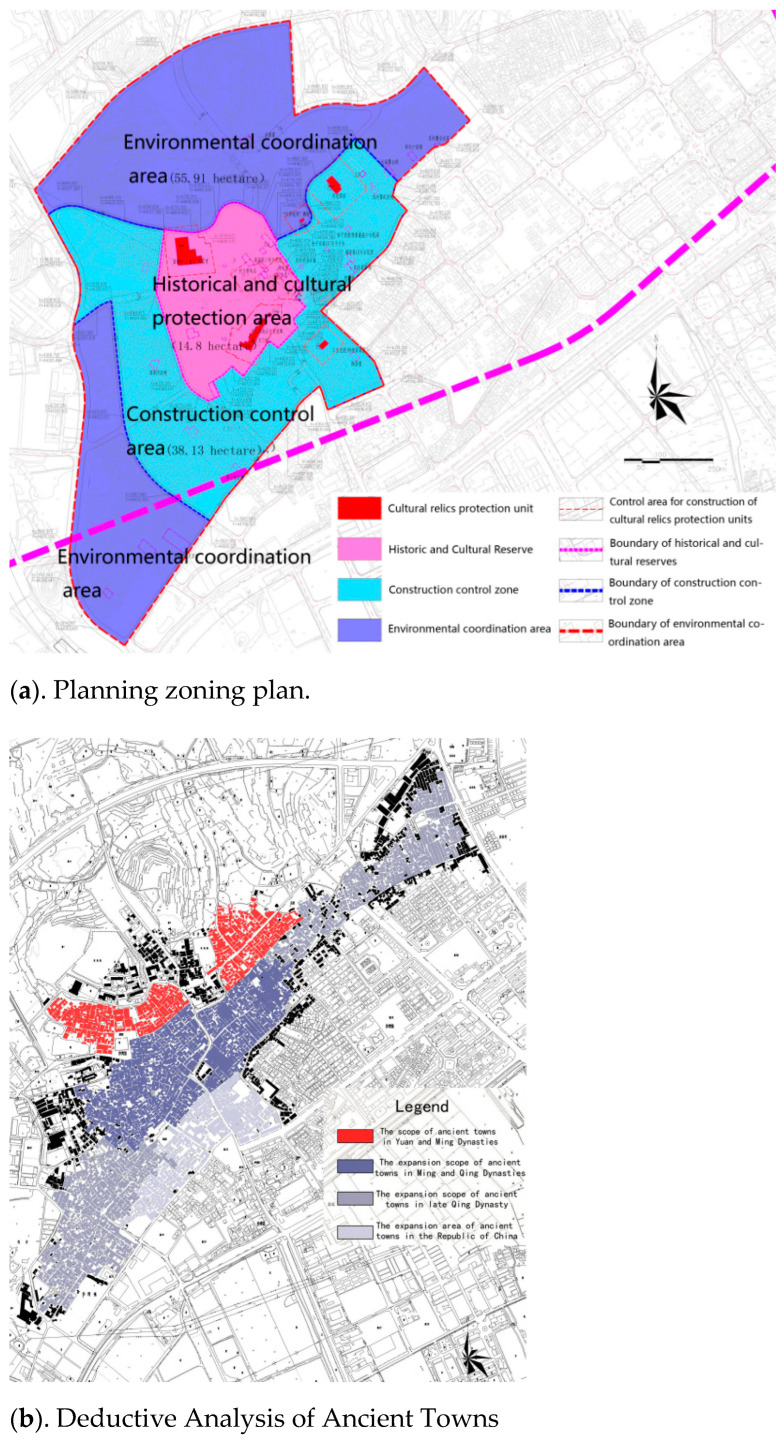
Field Analysis Diagram (**a**). Planning zoning plan. (**b**). Deductive Analysis of Ancient Towns.) (Source: [20]).

First, the radiation effect of cities on ancient towns should be reduced on a macroscale. The Shawan Historical and Cultural Reserve, with an area of 108.9 hectares, is planned, and its scope includes west of Shawan Avenue, north of Zhonghua West Road, east of Shaxi Road, and the area of the mountain in the north of the town. The overall planning of the Shawan Ancient Town is divided into three levels, namely, “Historical and Cultural Core Protection Zone, Construction Control Zone, and Environmental Coordination Zone” (Figure 6a). The core historical and cultural protection area is a relatively well-preserved village area formed during the Ming and Qing dynasties, with Liu Gengtang as its center, including traditional businesses and traditional residential culture with Anning West Street, Chepi Street, and Chengfangli as the main body. The construction control zone shall be the village environment within the urban purple line (Figure 6a). The environmental coordination area is the overall landscape level of one core, two streets, and three districts in this town other than the urban purple line protection and the construction control areas. Furthermore, the incremental changes in the spatial structure of settlements in different areas are determined, and the protection level of each region is clarified, following the development history of ancient towns through the Yuan, Ming, Qing Dynasties, and Republic of China (Figure 6b).

### 4.2. Building Viewing Sequences and Compiling Renewal Networks

(1) Tour planning. The protection and renewal of the town follow the central hierarchical structure of “ancestral hall-square-main street-alley-courtyard”. It revolves around the historical information and relic materials in the narrative text, forming a network of distinct and focused organizational structures with core plaques, corridors, and nodes as the basic elements. Meanwhile, two sightseeing routes are planned on the basis of contextual clues.

Time travel: West Square–Liugengtang–Ping’an Lane–Dama Lane–Wenfeng Pagoda–Jinwei Lane–Anning West Street–Sanren Hall–Taishi Lane–Guanxiangli Street–West Square.

**Figure 7:**
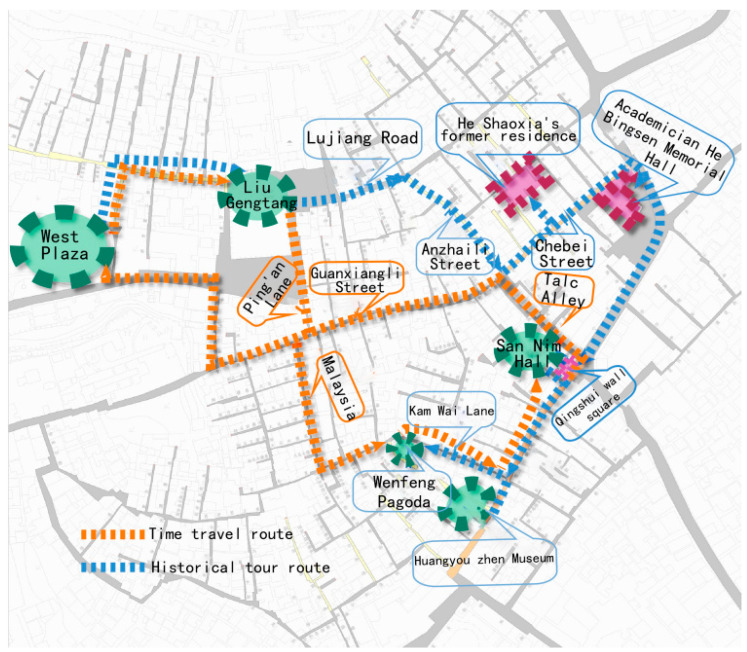
Narrative clues and tour route. (Source: the authors.)

Historical tour: West Square–Liugeng Hall–Lujiang Road–Anzhaili–Chepi Street–Former Residence of He Shaoxia–Academician He Bingsen Memorial Hall–Qingshuijing Square–San Ren Hall–West Anning Street–Huangyou Town Yabai Art Museum–Jinwei Lane–Wenfeng Pagoda (Figure 7).

**Figure 8:**
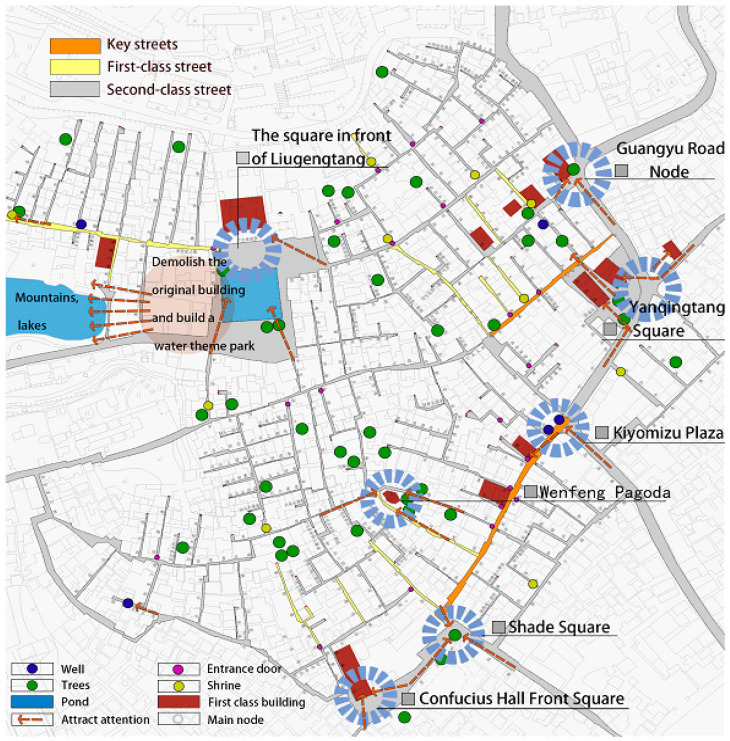
Key transformation node diagram. (Source: the authors).

**Figure 9:**
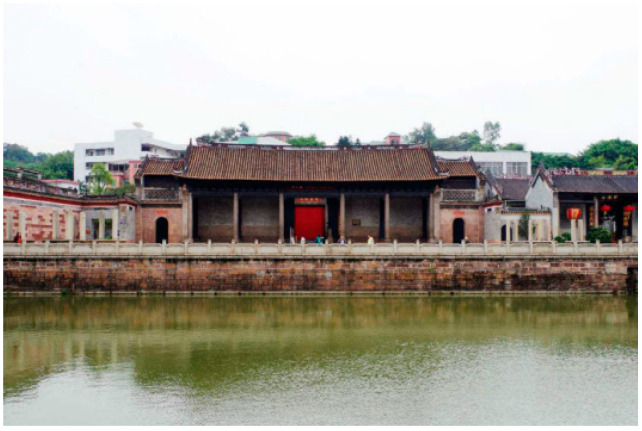
Liugengtang Square. (Source: the authors).

**Figure 10:**
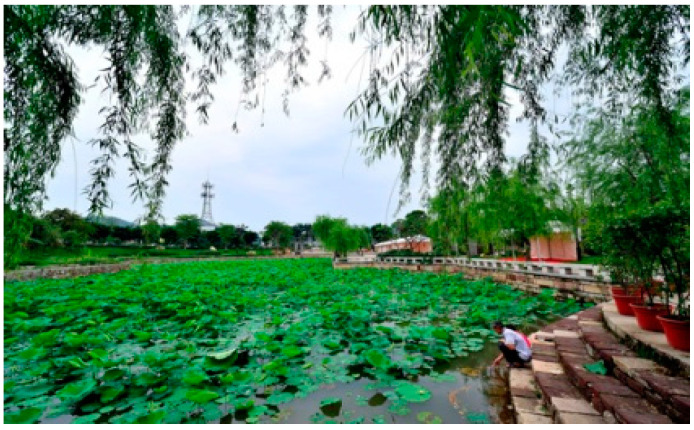
West Square. (Source: the authors.).

(2) Core expansion. A landscaped plaza with a Liugengtang ancestral hall and a feng shui pond as the center is created. Concurrently, the connection between the ancestral hall square and the waters in the west are expanded, and the discordant new structures on the site are removed. The spatial pattern relationship between this town and the surrounding mountains, forests, and rivers is protected through the spatial integration construction of the hall square and pond area, meeting the functional needs of public recreation and various folk activities, and expanding the scope of historical landscape patterns (Figure 8).
(3) Street grooming. The first-class main streets, “Anning Street, Chepi Street, and Daxiangyong Road”, and the second-level lanes, “Chengfangli, Anzhaili, Dunhouli, Guanxiangli, Cuizhuju, and Yazhongfang”, and other “one dwelling, three lanes, and thirteen miles”, are selected as the key points for renovating the streets. According to the different levels of streets and alleys, corresponding rectification efforts are implemented, and the repair and transformation of important historical buildings in each street and alley are highlighted. Simultaneously, several squares are built at the intersections of streets and lanes, and the local boundaries and fields of the town are appropriately dredged to meet modern functional requirements (Figure 9 and Figure 10). The interior space alleviates the overly dense streets and alleys. Following the principle of only reducing and not increasing, some uncoordinated buildings are selectively relocated, and the reserved homestead space is transformed into a greening and rest node to enhance the recreational greening area of the interior space.

### 4.3. Clear Eras: Standard Quantitative Adjustment of Interface Morphology

If the mechanical application of regional traditional forms and systems is easy to ignore, the special value of residential styles in different periods results in the homogeneity of architectural characteristics in the entire village and town. Therefore, the concept of “clear eras” is applied in the renovation of street and lane facades, which organically preserves the construction forms of different historical stages and places them on the original interface, showing the time imprint of the process of time continuation and derivation.

**Figure 11:**
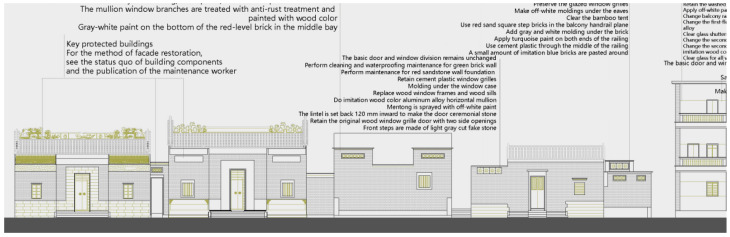
Facade renovation of Chepi Street (Source: [20]).

First, the renovation is classified on the basis of the building type and current condition, and corresponding renovation measures are planned for cultural relic, historical, and general buildings, respectively (Table 2). Then, the number and types of key buildings are counted, and independent remediation files for various types of buildings are established (Table 3). Some residential buildings with historical characteristics from the Republic of China, the founding of the People’s Republic of China, and the Cultural Revolution are preserved on the basis of repairing the old as before, while protecting and repairing the cultural relics of the Ming and Qing dynasties. They are also refurbished or repaired to enhance their use function according to their current conditions. For modern tiled houses that are not in harmony with the environment, measures are taken to replace their facade skin and symbolic components to complete the integrity of the overall street and alley construction style. Finally, a pattern of juxtaposed historical buildings of blue brick, red brick, gray sand, and the stone-rice houses is formed, maintaining the sense of real life and diversity of traditional village spaces (Figure 11).

**Figure 12:**
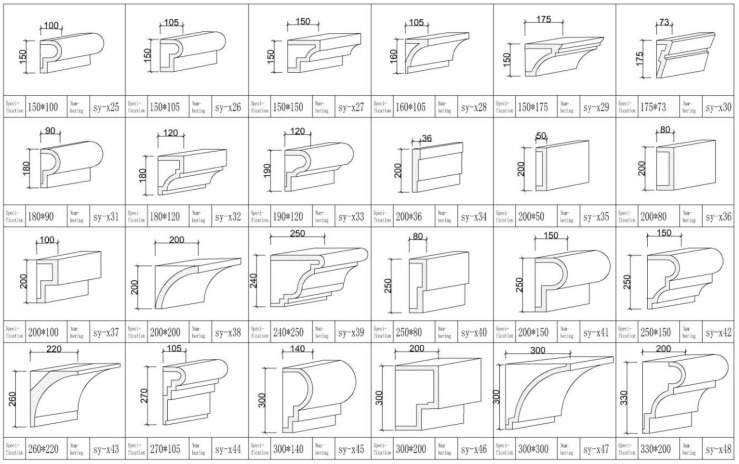
Line style series. (Source: the authors.).

**Figure 13:**
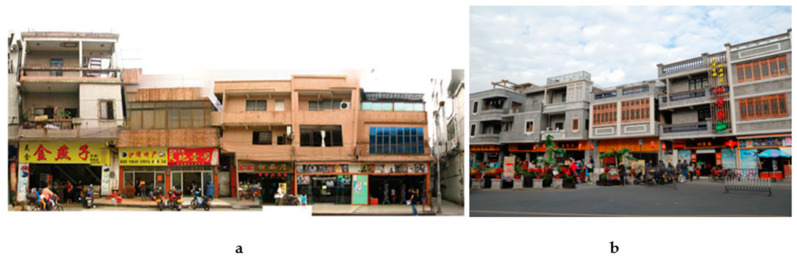
Renovation of Daxiangyong Road (Source: [31]) (**a**). Part of the east side of Daxiang Chong (before reconstruction) (**b**). Part of the east side of Daxiang Chong (after reconstruction).

**Figure 14:**
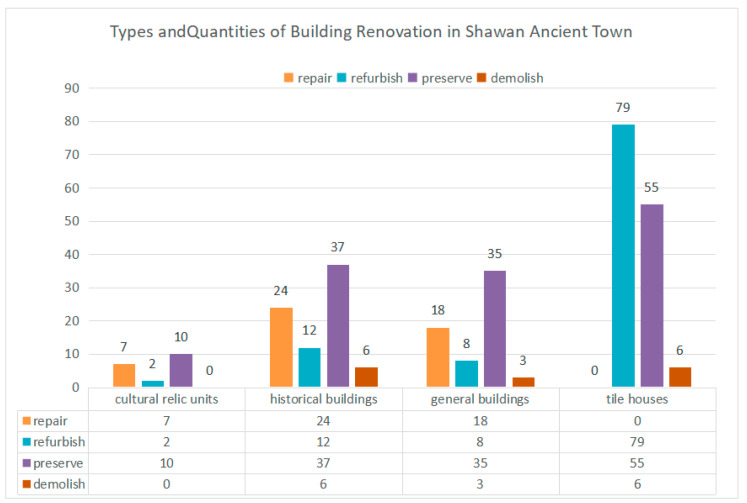
Types and quantities of building renovation in Shawan Ancient Town. (Source: the authors.).

Second, a standardized construction paradigm is established in response to the large and complex number of buildings. The decorative features of traditional Cantonese houses are extracted to form a systematic set of decorative lines and component styles (Figure 12). Corresponding lines and components are matched for various types of renovated buildings, and the similarities and differences between the building units are grasped flexibly to form a standardized renovation paradigm that effectively responds to cluttered interfaces. For example, Daxiangchong Road is the transition area between the Shawan Ancient Town and the surrounding cities; almost all the streets and alleys are modern tiled buildings, exerting a significant impact on the traditional atmosphere of the town. Renovations respect the owner’s usage habits and functional requirements to renewal the facade while not changing the original building structure. First, the blue brick skin of the tile house and the style of doors and windows are replaced, and foot restraints and decorative components are added to the doors, windows, balconies, and guardrails. Meanwhile, the contrast between the flat roof and the sloping roof, as well as the difference in the form of the wooden doors and windows and the Manchurian windows, is used to highlight the changes in the details of each house while maintaining the overall unity (Figure 12 and Figure 13). After many rounds of material and shape selection, the interface style of Daxiangyong Road plays a good transitional role in the connection between the new city and the ancient town (Figure 14).

### 4.4. Plaque Symbiosis: The Reappearance of Historical Memory of the Place

**Figure 15:**
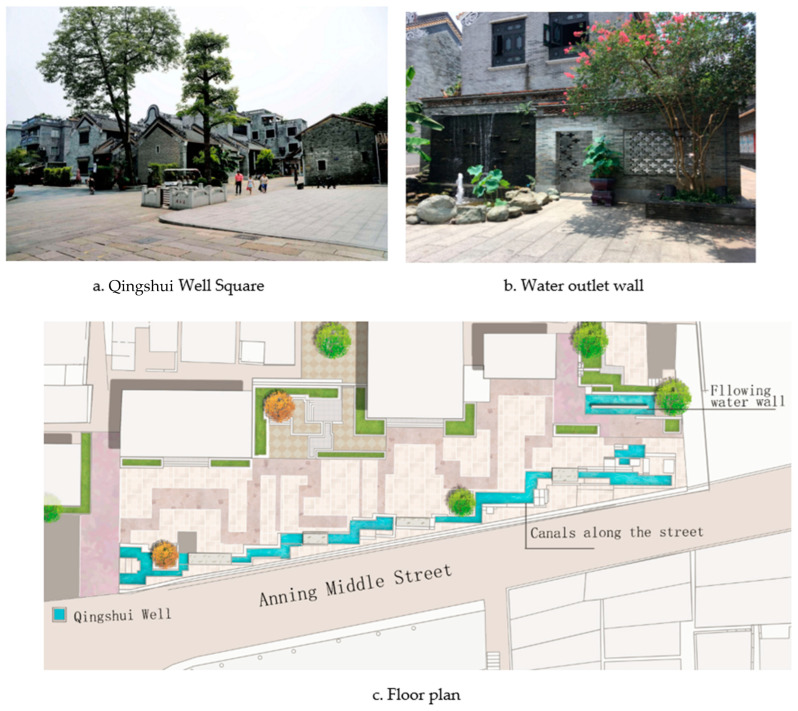
Qingshui Well Square. (Source: the authors.).

At the intersection of Anning Middle Street and Daxiangyong, a historical relic built in the Ming and Qing dynasties, Qingshui well can be found in the south of the Shawan Ancient Town (Figure 15a). According to the historical records of Shawan, there was a large alley gushing water through Anning Middle Street in ancient times [20]. The Qingshui well at this intersection has become a symbol of Shawan’s centuries-old water town culture and history. Many shops surround Qingshui well, and the business atmosphere is strong. The design takes water cultural memory as the narrative theme to create a plaza for public recreation. The water outlet is designed to create a landscape of pools and stacked water walls at the starting point of the east end (Figure 15b) The texture symbols of the wok ear wall of traditional Cantonese dwellings are integrated into the landscape wall and sidewalk. One side is connected with a belt-shaped canal leading to the Qingshui well, and the canal expresses the intention of the river with a “folded” moving line, which is a metaphor for the vertical and horizontal scenes of the waterways in Shawan in ancient times. Meanwhile, the canal runs parallel to the road, and the largest square usable area is reserved, extending from the water outlet to the canal of the Qingshui well to form an oriented landscape sequence and a waterscape narrative topic (Figure 15c). The water channel is a metaphor for the image of the historical water system in a modern form and has a resonance and symbiosis with the real historical relics of the Qingshui well. 

**Figure 16:**
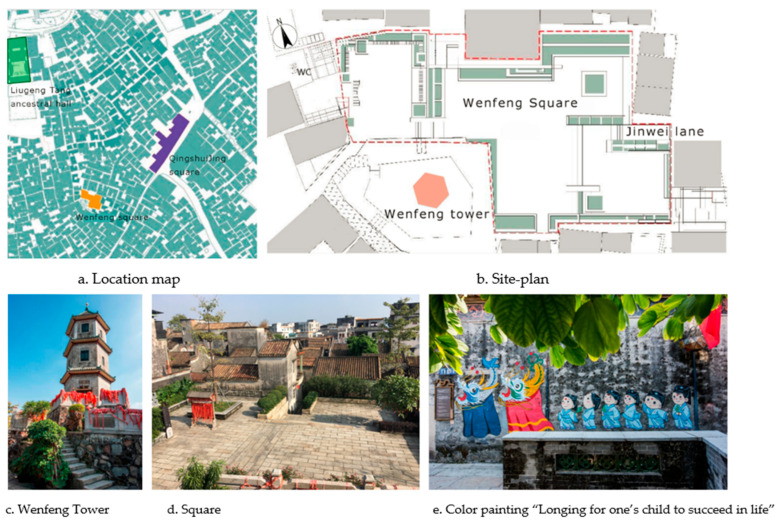
Wenfeng Square transformation (Source: [31]).

There is a Wenfeng Tower (approximately 300 m from Liugeng Hall) in the southeast of the Shawan Ancient Town, which is an architectural form used to promote cultural movement in feng shui planning while guarding the “water mouth” of the Shawan Ancient Town (Figure 16a,c). The Wenfeng Tower is a brick and wood structure of six hexagonal three-story blue brick towers, approximately 20 m high, with the first floor dedicated to the “Wenchang” idol, the second to the “Guan Di” idol, and the top to the “Kuixing” idol, where rural children come to worship the gods before they are “off to school”.

To expand the space and view of the three sides of the Wenfeng Tower, the two old houses next to the tower were demolished to form a cultural landscape that echoes Liugeng Hall from a distance. The square is divided into three small platforms by elevation difference, with each platform having a drop of approximately 1 m (Figure 16b) The site uses numerous local “wax gourd strips” stones to build the ground, flower pool, and steps. The primitive material texture creates an ancient atmosphere of historical accumulation of the site (Figure 16d) and realizes the patch regeneration centered on the Wenfeng Tower. Additionally, the wall beside it is painted with a painting of “longing for one’s child to succeed in life” (Figure 16e) to elaborate the topic narrative of Wenfeng Tower’s implied meaning, thereby strengthening the cultural spirit of the place and integrating the elements of the theme, forming a synthetic expression of the combination of traditional and modern themes.

### 4.5. Fragment Picking: Ritual Embedding of Landscape Elements

**Figure 17:**
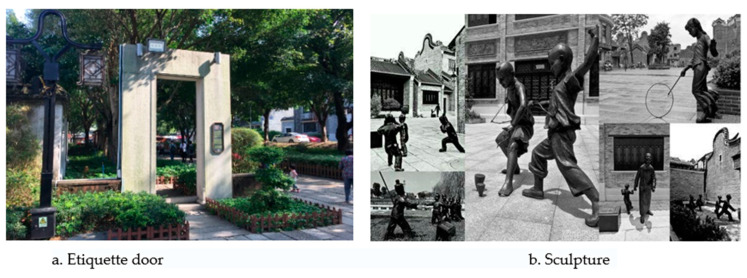
Diversified media narrative. (Source: the authors).

There are many small, scattered node spaces in villages and towns, such as village entrances, street corners, room vacancies, and quays. The ritualized narrative of these point-like spaces strengthens the recognizability of living memory. For example, Shimen landscapes with similar installation characteristics are designed at the entrance, middle, and end sections of Shawan Ancient Town, such as the gate of etiquette, the gate of loyalty and filial piety, and the gate of life. The triple gate interacts with human behavior in isolated, discontinuous, and diverse forms, resulting in a landscape installation-implying life philosophy (Figure 17a). Second, random paintings are drawn on the corners of the houses in the streets and alleys, showing the creative theme that fits the place and generating a narrative experience of modern nostalgic aesthetic feelings. The landscape sketches and graffiti series integrated into the streets and alleys have become a narrative thread that juxtaposes with the main thread, constructing a dialog between the aesthetics of modern art and the regional context (Figure 17b).

**Figure 18:**
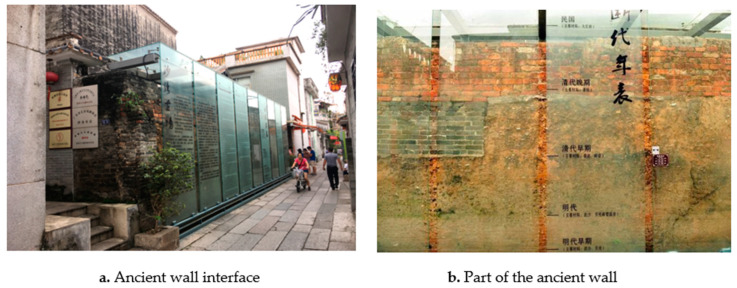
Protection of Shawan Ancient Wall. (Source: the authors).

There is a residual wall built in the Ming Dynasty (Figure 18a) in the north of this ancient town, Wenlinfang Street–Lujiang Zhoudao Lane, with a height of 4 m and a length of 20 m. The wall has been built and changed over several hundred years. The internal materials are also integrated into the materials of various historical periods. From bottom to top, they are mixtures such as sand, shells, and broken porcelain in the early Ming Dynasty; yellow mud and broken porcelain in the early Qing Dynasty; blue bricks in the late Qing Dynasty; and red bricks in the Republic of China. It has become a historical symbol of Shawan’s traditional wall construction development. It is designed with a tempered glass curtain wall outside the original residual wall to form a protective surface (Figure 18b). The glass surface records its historical background, material composition, and cultural value in words, making it a “historical memory highlight” to verify the construction and changes of Shawan Ancient Town in the past dynasties.

**Figure 19:**
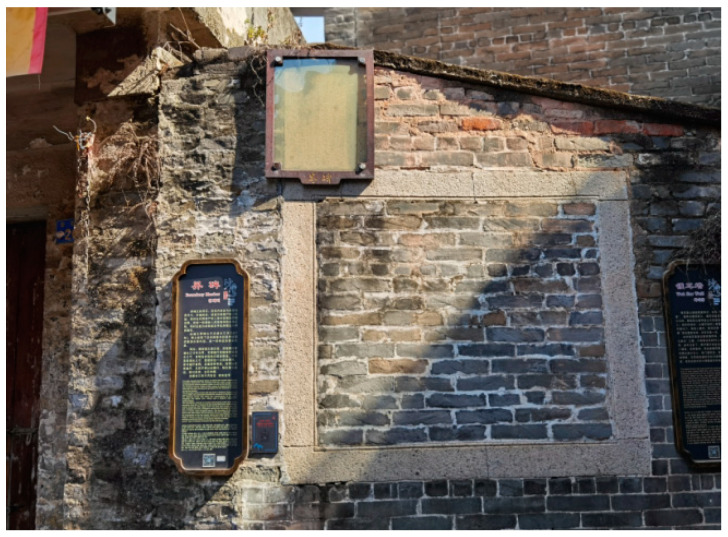
Boundary monument wall. (Source: the authors).

**Figure 20:**
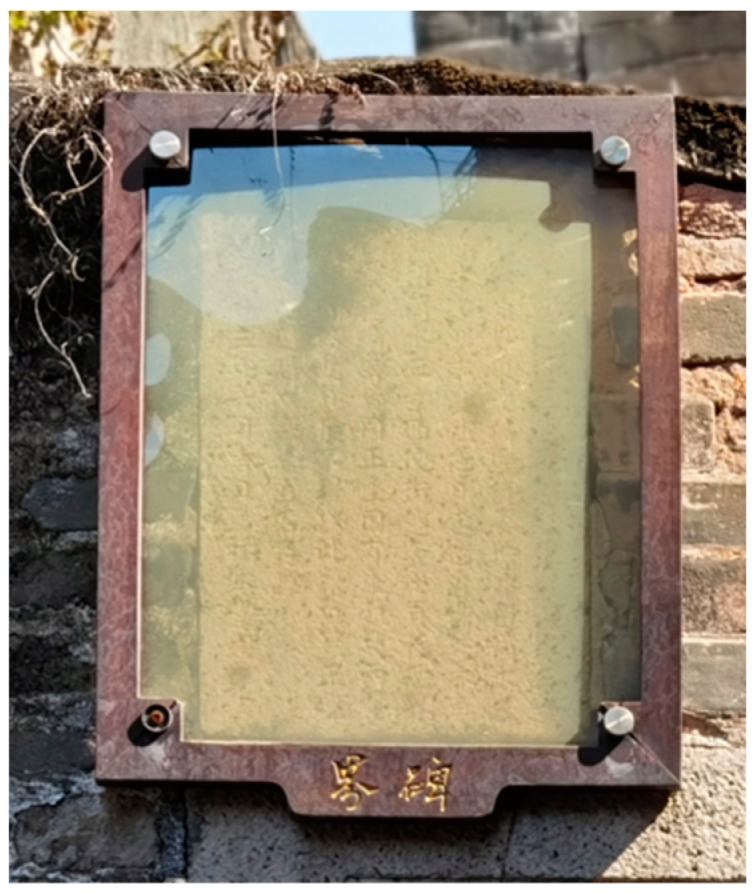
Front of the boundary monument. (Source: the authors).

At the interface of the villages and towns, there are several left boundary markers (also known as boundary stones, Figure 19 and Figure 20). Most of its content is to clarify the size of the boundary and the four sides of the outer wall or to declare the human rights of housing property. For example, a land certificate embedded in the wall can also guide relatives and friends to find the owner of the house. Its inscription is as follows:

There are still three and a half inches of dripping water on the back wall as the boundary in the main seat of Suijitang’s house, and three inches of dripping water is left on the outside of the rear eaves of the east side hall. It is stated that Ji Qingzu will build the watch wall in the future, and there must be three inches of dripping water outside. A small page at the back of the west side street was the same as that on the side hall. There are side halls on both sides due to a dispute over the main seat. One should not use this as an excuse in the future, and one will be suspicious of sitting in the right seat. The fear that there is no evidence, and the stone is preserved. (Twenty-three years in June auspicious day in Guangxu Dynasty, Shu Bentang.)

The design is protected using a clear glass cover, and the display board is hung below the boundary monument as a functional interpretation. These walls and boundary markers at the corners of the settlement serve as scattered narrative clues, expanding the narrative boundary in form, scale, and orientation and activating the narrative function of more detailed nodes. The semantic information of multiple clues evolves and is superimposed in the field with the development of each viewing path, constituting delayed reading and time series aesthetics of the context of the field [32].

## 5. Discussion 

Tschumi once emphasized the importance of events, people’s subjective experience, and perception in conveying cultural significance in the entire process of spatial narrative [33]. The core task of the spatial narrative is to present the historical information and cultural significance of objects, fields, and events and to spread, transform, and create them among speakers, users, and places [34]. 

The previous studies have proposed a variety of renewal modes for the renewal of traditional villages in China, such as the “acupuncture renovation mode" [35], that is, the protection and renewal path around the heritage scattered in the rural space. It has also proposed an “authentic renovation mode” [6], which concentrates on the authentic patterns and boundary of the evolution process of the countryside thus providing realistic clues for rural protection planning. There is also another mode of “overall transformation mode” [36], which means the unified construction mode of the rural interface to maintain the integrity of the style. These modes mainly focus on the planning ideas of villages and the construction skills of formal symbols. However, as historical and cultural heritage resources, the value of traditional villages is also reflected in the excavation and representation of their collective memory and historical information, which are invisible and acted as the soul of traditional villages. Therefore, using the narrative theory and methods can more expose that and show the recessive connotation behind material appearance of the village, which presents the uniqueness, interest, and identity of spatial semantics in a kind of field complexity. Furthermore, the essence of its transformation is to build an interactive rooted relationship between users, space, and cultural information [37].

## 6. Conclusions

(1) The protection and renewal of ancient villages should be realized on multiple levels. At the macrolevel, the protection planning level is constructed around the core area, the buffer area for style protection is set from the periphery of it, and the intensity of renewal and transformation is set from the inner space subarea. At the mesolevel, the narrative uses the central narrative paradigm of structuralism, forming a central narrative sequence centered on multiple ancestral halls, structures, and squares. Meanwhile, the spatial deduction process is stated in the historical linear development motif, and the contextual inheritance under the contrast between the old and the new is explored in the integrity of the interface style. At the microlevel, the juxtaposition of multiple narrative clues from the center, corridor, plaque, and node deepens the narrative situation. It applies the “seeking a change in the whole” method, seeks the individual changes of interface components in repetition and listing, and integrates rich historical and differentiated aesthetic styles.
(2) Memory and events are the sources of the rural field narrative and space is the carrier of narrative existence. Rural narrative enriches new implicit ideas and themes, providing viewers with a new experience of space, plot, and historical activities while writing historical stories and collective memories in the context of rural society.
(3) The protection and renewal of Shawan Ancient Town is first rooted in the original historical information, cultural heritage and the pursuit of the integrity of historical features, then corresponds to the information mining of “culture–space” and “time memory”. It constructs the framework and methods for the renovation of multiple unit places, corridors, and residential buildings in the ancient town, and expand the display path for the memory mining and event reappearance of traditional villages. This updated mode maintains the cultural context of ancient villages in a real way and effectively avoids the destructive construction with large numbers of fake antiques. However, in the process of renewal, a large amount of capital, long-term human maintenance and time costs are required. Therefore, this mode is more suitable for traditional villages with profound history and culture, while it is necessary to actively balance practical contradictions to generate a transformation path according to local conditions and functional requirements for ordinary villages with weak cultural accumulation.

## Figures and Tables

**Table 1:** Classification of narrative themes in Shawan Ancient Town.

Classification	No.	Theme	Narrative Construction	Space Location
Character commemoration	1	He Shaoxia	He Shaoxia’s former residence	Huiyan Lane, Chebei Street
	2	He Binglin	Academician He Binglin Memorial Hall	Anning West Street
	3	Huang Youzhen	Huangyou Town Thuja Art Museum	Anning West Street
Secular life	1	Sand bay music	Shawan Guangdong Music Hall	Anning West Street
	2	Floating color art and lion-awakening dance	San Nim Hall	Anning West Street
	3	Three kinds of carving and one kind of grey sculpture		Each node
	4	Folk games	Installation sculpture	
Spiritual beliefs	1	Liugeng Ancestral Hall, Yanqing Hall, and Wang Ancestral Hall	The square in front of Liugengtang hall and Yanqingtang hall	Shawan North Village and West Village
	2	Yuxu Palace, Ancient Temple of Emperor Wu, and Guanyin Temple	Yuxu Palace	Shawan North Village
	3	Wenfeng Pagoda	Wenfeng Pagoda Square	Shawan North Village
Historical relics	1	Shawan Ancient Wall	Ancient wall landscape	Wenlinfang Street
	2	Qingshui well and original river channel	Kiyomizu Plaza	Anning West Street

**Table 2:** Renovation mode of ancient town buildings.

Type	Transformation Method			
	Repair	Renovation	Reserve	Demolish
Heritage building (building type before the Republic of China)	Daily maintenance, protection and reinforcement, and key repairs. Implement the principle of “not changing the original state of cultural relics” and truthfully reflect historical relics.	If the internal structure is seriously damaged, functional replacement will be performed to reuse the building, the internal structure will be replaced, and the internal space pattern will be reset.	For the cultural relic units with better status, they will be maintained as they are.	No
Historical buildings(red brick, gray-sand, and stone-rice houses in the 1950s and 1990s)	For historical buildings that can reflect the characteristics of various historical periods, the original characteristics and basic materials are not changed, and raw materials are used to restore them.	For historical buildings whose exterior facades are severely damaged and internal functions decline, necessary renovations should be made according to specific conditions, such as adding toilets and improving interior space.	Do not interfere with well-functioning historic buildings.	Demolish individual dilapidated houses with severe functional degradation and difficulties to maintain.
General building (modern tile room)	Repair or material replacement of partially damaged surfaces of buildings.	For new ceramic tile buildings with great influence on style and good quality, measures, such as reducing the number of floors, replacing components, and changing facade materials, are used.	Buildings that have no obvious conflict with style or that do not affect the style program should be reserved.	For buildings that are difficult to deal with after renovation (such as dilapidated and temporary houses), the demolition method is used.

**Table 3:** Housing repair archives.

Building number	F0105	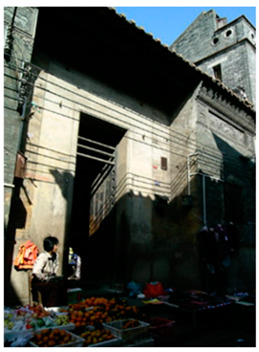	
Building name	12 Anning West Street		
Protection type	Maintenance building		
Building type	Low-rise single-family house		
Building floors	One layer		
Structural materials	Masonry		
Protection measures	Main building	Facade	For the repair method, please refer to the current situation of building components and the table of maintenance methods. Protect and repair, according to the principle of repairing the old as before. The repaired and reinforced parts should be slightly different in color from the original components to maintain the authenticity of the building.
		Interior	For the repair method, please refer to the current situation of building components and the table of maintenance methods. The interior space is demolished, and the added part is replaced to the original spatial pattern. Reinforcement and maintenance should follow the principles of necessity and reversibility.
		Interior	The original space pattern is restored, and the function can be adjusted.
	Environment	Greening	Increase garden greening.
		Other	N/A

## Data Availability

The data presented in this study are available on request from the corresponding author. The data are not publicly available due to the restriction on privacy.
